# Alcohol, psychoactive substances and non-fatal road traffic accidents - a case-control study

**DOI:** 10.1186/1471-2458-12-734

**Published:** 2012-09-03

**Authors:** Stig Tore Bogstrand, Hallvard Gjerde, Per Trygve Normann, Ingeborg Rossow, Øivind Ekeberg

**Affiliations:** 1 Emergency Department, Division of Emergencies and Critical Care, Oslo University Hospital, Ullevål, Oslo, N-0407, Norway; 2 Department of Research and Development, Division of Emergencies and Critical Care, Oslo University Hospital, Ullevål, Oslo, N-0407, Norway; 3 Norwegian Institute of Public Health, Division of Forensic Medicine and Drug Abuse Research, PO Box 4404, Nydalen, Oslo, N-0403, Norway; 4 Norwegian Institute for Alcohol and Drug Research, PO Box 565, Sentrum, N-0105, Oslo, Norway; 5 National Centre for Suicide Research and Prevention, University of Oslo, Sognsvannsveien 21 Bygg 12, 0372, Oslo, Norway; 6 Department of Acute Medicine, Oslo University Hospital, Ullevål, Oslo, N-0407, Norway

**Keywords:** Alcohol, Case–control, Emergency treatment, Injury, Psychoactive substances, Road traffic accident

## Abstract

**Background:**

The prevalence of alcohol and other psychoactive substances is high in biological specimens from injured drivers, while the prevalence of these psychoactive substances in samples from drivers in normal traffic is low. The aim of this study was to compare the prevalence of alcohol and psychoactive substances in drivers admitted to hospital for treatment of injuries after road traffic accidents with that in drivers in normal traffic, and calculate risk estimates for the substances, and combinations of substances found in both groups.

**Methods:**

Injured drivers were recruited in the hospital emergency department and drivers in normal conditions were taken from the hospital catchment area in roadside tests of moving traffic. Substances found in blood samples from injured drivers and oral fluid samples from drivers in moving traffic were compared using equivalent cut off concentrations, and risk estimates were calculated using logistic regression analyses.

**Results:**

In 21.9% of the injured drivers, substances were found: most commonly alcohol (11.5%) and stimulants eg. cocaine or amphetamines (9.4%). This compares to 3.2% of drivers in normal traffic where the most commonly found substances were z-hypnotics (0.9%) and benzodiazepines (0.8%). The greatest increase in risk of being injured was for alcohol combined with any other substance (OR: 231.9, 95% CI: 33.3- 1615.4, p < 0.001), for more than three psychoactive substances (OR: 38.9, 95% CI: 8.2- 185.0, p < 0.001) and for alcohol alone (OR: 36.1, 95% CI: 13.2- 98.6, p < 0.001). Single use of non-alcohol substances was not associated with increased accident risk.

**Conclusion:**

The prevalence of psychoactive substances was higher among injured drivers than drivers in normal moving traffic. The risk of accident is greatly increased among drivers who tested positive for alcohol, in particular, those who had also ingested one or more psychoactive substances. Various preventive measures should be considered to curb the prevalence of driving under the influence of psychoactive substances as these drivers constitute a significant risk for other road users as well as themselves.

## Background

Psychoactive substances are prevalent in emergency department samples of injured drivers, and several studies have found high prevalence rates not only of alcohol but also of medicinal and illicit drugs [1-3]. Studies of drivers in normal moving traffic have shown lower prevalence rates [4-6]. In line with this, case–control studies have demonstrated that presence of psychoactive substances in drivers is associated with an elevated risk of traffic injuries [7-10]. The elevated risk can be explained by the impairing effects of psychoactive substances on driving ability (see for instance [11]). In this study we apply a case–control design to address psychoactive substance use and non-fatal traffic injuries.

Several types of methodological weaknesses hamper many of the case–control studies that have assessed the relative risk of psychoactive substance use for traffic injuries. These comprise non-representative samples of controls (e.g. other patients) and non-equivalent biological samples for cases and controls ([7]). In an ideal situation controls should be random drivers in normal traffic and blood samples should be collected from both cases and controls, as blood is the biological sample that best reflects the drug concentrations in the central nervous system (CNS). Earlier case–control studies of psychoactive substance use and non-fatal traffic accidents have not been performed in an ideal situation. Mura and co-workers used blood samples from both cases and controls, but the controls were not random drivers in normal traffic [9], which implies a possible selection bias in the control group. In the study by Movig and co-workers controls were sampled from drivers in normal traffic, but blood samples were compared with urine samples [8]. As psychoactive substances have longer detection times in urine than in blood [12] their presence in urine does not necessarily reflect impaired driving. Comparisons of blood and urine samples can therefore bias the relative risk estimates downwards.

The ideal situation with bloods samples from controls in normal moving traffic is, however, very difficult to obtain. It is costly to obtain a sufficiently large sample of random drivers in normal traffic and it is difficult to collect blood samples in normal moving traffic. Drivers may be reluctant to participate in roadside studies and to give blood samples, even with monetary incentives [13]. Encouraging high levels of participation is very important as the total prevalence may be as low as 4.5% or less for both alcohol and other psychoactive substances among drivers in moving traffic [4-6].

One way of overcoming the problem with collection of blood samples in random drivers is to use oral fluid for drug screening in moving traffic. Collection of oral fluid has proved successful in random drivers, with low refusal rates [5]. Oral fluid can be compared to blood samples as both specimen types are positive for drugs for the same average length of time after intake of a single dose. The distribution of drug concentrations in oral fluid reflects the distribution of drug concentrations in blood in a population [14]. Comparisons between blood samples and oral fluid samples can be made if equivalent cut-off concentrations in blood and oral fluid are used [14]. One previous case–control study has compared psychoactive substances in cases and random driver controls applying comparable biological samples (blood and oral fluid) to assess relative risk estimates for traffic injuries [7]. While these estimates were for fatal traffic injuries we have in this study applied the same study design with random driver controls and comparable biological samples to assess relative risk estimates with respect to non-fatal injuries.

Combinations of drugs are prevalent among injured drivers [2,3,15] while combinations in moving traffic are rare [5]. Some studies suggest that combinations of various psychoactive substances constitute a particular increased risk of traffic injuries [8,11]. In line with this, a recent Norwegian case–control study of fatal road traffic accidents found few single substance cases in the accident group but a major increase in risk for drivers with combinations of psychoactive drugs [7]. It therefore seems particularly important to assess the impact of multiple psychoactive substance use on traffic injuries.

The aim of our study was to compare the prevalence of alcohol and psychoactive drugs in drivers admitted to hospital for treatment of non-fatal injuries after road traffic accidents with prevalence in drivers in normal traffic, and to calculate odds ratios for single and multiple substance use.

## Methods

### Study design and setting

This study has a case–control design. Cases were patients admitted to the regional trauma centre for treatment of injuries after driving a car involved in an accident. Controls were a large sample of drivers from normal moving traffic in the hospital catchment area. The hospital is the trauma centre for the south-eastern part of Norway covering both urban and rural areas and including the capital, Oslo. About 2.5 million people live in the catchment area of the trauma centre. The source population was drivers in moving traffic in the south-eastern parts of Norway.

### Description of cases

#### Inclusion of injured drivers

Drivers older than 18 year of age admitted to the emergency department at Oslo University Hospital, Ullevål, were included. Criteria for inclusion was informed consent from the patient or his or hers next of kin if the patient was not able to give an informed consent. If the patient was unavailable to give consent in the emergency department because of acute medical treatment or for other reasons, blood samples were obtained and the patients were asked to give informed consent later during the stay. If the patient refused to participate, was discharged before giving consent or for other reasons could not give an informed consent, the blood sample was destroyed. No data was registered on non participants. The data was collected over a 12 month period from December 2007 to December 2008. Further details on study inclusion have been published in an earlier paper [1].

#### Data collection

All patients included in the study filled in a questionnaire comprising gender, age, time of accident and type of accident. If the patient was not able to fill out the questionnaire, for example because of injury, an emergency department nurse assisted them. Blood samples were drawn, together with diagnostic blood samples and sent to the Norwegian Institute of Public Health (NIPH) for analysis. The time of blood sampling was recorded and samples taken more than six hours after injury were excluded from this study.

#### Blood sample analysis

The blood samples were analyzed according to the forensic toxicology analytical programme using two different methods for screening and confirmation analyses of the drugs. The samples were first screened for amphetamines, cannabis, cocaine metabolites and opiates by an immunological method [16]. Screening for other drugs was carried out using high-performance liquid chromatography with mass spectroscopy detection (LC-MS) [17]. Then, all drug findings were confirmed and quantified using gas or liquid chromatography with mass spectroscopy detection (GC-MS or LC-MS) [17-20]. An enzymatic dehydrogenase method was used for ethanol analysis [21]. Analysis was carried out for a total of 20 different substances, constituting all impairing drugs on the Norwegian market which have been shown to be linked to increased accident risk [22]. Analytical cut-off values corresponding to the cut-off used in the DRUID project (http://www.druid-project.eu) were used, as described in Table 1 [23].

**Table 1:** Prevalence and cut-off values in whole blood and oral fluids (n = 5401)

	**Case group (Injured drivers (n = 96)**		**Control group (Moving traffic n = 5305)**	
**Substance**	**Cut-off in whole blood**	**Prevalence% (n)**	**Cut-off in oral fluid**	**Prevalence% (n)**
***Any substance***		***21.9 (21)***		***3.2 (169)***
***Alcohol***	***0.02 g/kg***	***11.5 (11)***	***0.02 g/kg***	***0.3 (17)***
***Stimulant drugs***	***ng/mL***	***9.4 (9)***	***ng/mL***	***0.5 (25)***
Methamphetamine	20	5.2 (5)	410	0.2 (13)
Amphetamine	20	4.2 (4)	360	0.1 (7)
Benzoylecgonine	50	4.2 (4)	95	0.2 (10)
Cocaine	10	2.1 (2)	170	0.1 (4)
MDMA	20	0	270	0.0 (1)
MDA	20	0	220	0
***Benzodiazepines***	***ng/mL***	***7.3 (7)***	***ng/mL***	***0.8 (43)***
Diazepam	57	3.1 (3)	2.0	0.5 (25)
Clonazepam	10	3.1 (3)	1.7	0.1 (6)
Alprazolam	10	3.1 (3)	3.5	0.0 (1)
Flunitrazepam	1.6	2.1 (2)	0.3	0.1 (3)
Oxazepam	50	0	13	0.1 (6)
Nitrazepam	7.0	0	2.0	0.1 (5)
***Cannabis (THC)***	***1.0 ng/mL***	***3.1 (3)***	***27 ng/mL***	***0.7 (39)***
***Z- Hypnotics***	***ng/mL***	***2.1 (2)***	***ng/mL***	***0.9 (49)***
Zopiclone	10	2.1 (2)	25	0.9 (49)
Zolpidem	37	0	10	0
***Opiates***	***ng/mL***	***1.0 (1)***	***ng/mL***	***0.3 (18)***
Methadone	10	1.0 (1)	22	0.1 (3)
Morphine	10	1.0 (1)	95	0.0 (1)
Codeine	10	0	94	0.2 (13)
Heroin (6-MAM)	10	0	16	0.0 (1)
***Combinations with alcohol***				
Alcohol and other psychoactive substance(−s)		4.2 (4)		0 (2)
***Positive samples with no alcohol***				
1 non- alcohol substance		3.1 (3)		2.5 (132)
2 non- alcohol substances		4.2 (4)		0.3 (16)
3 or more non- alcohol substances		3.1 (3)		0.1 (4)

### Description of controls

#### Selection of drivers in roadside survey

The selection of drivers of cars and vans (reference group) was carried out in collaboration with the Mobile Police Service (MPS) in south-eastern Norway as a part of the DRUID project. Drivers were selected from April 2008 to March 2009 using a stratified multi-stage cluster sampling procedure. The first stage consisted of selecting regions in south-eastern Norway. The second stage covered selection of road sites, and dates and times for the sampling, while the third stage consisted of randomly stopping of cars and vans for routine control of breath alcohol or driving licence by the MPS. Participation was voluntary and anonymous. Our study team asked as many drivers as possible for participation in the project during two two-hour periods for each study day. Oral and written information was given to each driver; information leaflets were available in 12 languages. If informed consent was given, a sample of oral fluid was taken and a short questionnaire was filled in. The following data were recorded: gender, age, nationality of the driver, and type of vehicle. Drivers did not receive any reward for taking part in the survey.

#### Sampling of oral fluid

Samples of oral fluid were collected using Statsure Saliva Sampler™ (Statsure Diagnostic Systems, Framingham MA, USA). The oral fluid collection pad was placed under the tongue until the indicator turned blue or for five minutes in cases where the indicator did not turn blue within a shorter time. The vial was then capped and labelled with a bar code label identical to the bar code of the questionnaire. The sample was stored in a bag at approximately 5°C for a maximum of 6 hours, and then frozen at about −20°C.

#### Analysis of oral fluid

The amount of collected oral fluid was determined by weighing, and the dilution with buffer was taken into account when calculating analytical results expressed in g per kg or ng per ml of undiluted (native) oral fluid. Samples of less than 0.2 ml oral fluid were regarded as failed samples and therefore excluded.

Alcohol was analysed by an automated enzymatic method using alcohol dehydrogenase [21]. Drug concentrations in oral fluid-buffer mixtures were determined by liquid chromatography – tandem mass spectrometry [24]. Cut-off concentrations are presented in Table 1. Samples with drug concentrations above the linearity limits were diluted and re-analysed.

### Variables

Variables for cases and controls were compared as described in Table 2. Time of sampling and time of accident were compared according to time and season variables. The time intervals were defined to adjust for differences in accident rates at daytime and nighttime, and weekends and weekdays. Adjustment for season was made to compensate for the considerable variation in road conditions, e.g. winter with snow and ice. The time periods and seasons are described in Table 2. Comparisons of single substances and of specific combinations were carried out when they were present in both groups.

**Table 2:** Characteristics of cases and controls (n = 5401)

		**Cases% (n = 96)**	**Controls% (n = 5305)**	**Test statistic**^**a**^
*Demographics*				
**Gender**				X^2^ = 0.638
Male		76 (73)	72 (3839)	P = 0.424
Female		24 (23)	28 (1466)	
**Age Groups**				X^2^ = 24.507
17-24		23 (22)	9 (481)	P < 0.001
25-34		22 (21)	18 (946)	
35-49		28 (27)	36 (1890)	
> 50		27 (26)	38 (1988)	
*Sampling Time*				
**Season**				X^2^ = 21.276
1 Spring (Mar-May)		23 (22)	34 (1824)	P < 0.001
2 Summer (Jun-Aug)		22 (21)	13 (687)	
3 Autumn (Sep-Nov)		20 (19)	31 (1643)	
4 Winter (Dec-Feb)		35 (34)	22 (1151)	
**Day and time**				X^2^ = 47.346
1 Mon-Fri	04-10	21 (20)	11 (557)	P < 0.001
2 Mon-Fri	10- 16	22 (21)	27 (1439)	
3 Mon-Thu	16- 22	24 (23)	10 (525)	
4 Mon-Thu	22- 04	7 (7)	5 (256)	
5 Sat + Sun	04- 10	5 (5)	3 (180)	
6 Sat + Sun	10- 16	5 (5)	19 (1026)	
7 Fri-Sun	16- 22	7 (7)	18 (967)	
8 Fri-Sun	22- 04	8 (8)	7 (355)	

^a^Chi- square test.

Cut- off concentrations in blood was for all substances except two equal to those used in the DRUID project. The cut- off limits for flunitrazepam and diazepam was considered to be high and the legal limits for use when driving in Norway was used, for these drugs. The legal limits were equivalent to a blood alcohol concentration of 0.2 g/L [25]. Cut- off concentrations in oral fluid that were equivalent to those used for blood were calculated as described by Verstraete et al. [26].

### Statistical analysis

Comparisons of cases and controls were computed using bi-variate cross tables, and statistical significance was calculated with Pearson’s chi-square or Fishers exact test when expected count in a group was less than five. Adjusted odds ratios were computed using multivariate logistic regression. All analyses were made in PASW 17 (SPSS.inc, Chicago IL). The level of statistical significance was p **≤** 0.05.

### Ethics

The inclusion of the injured patients and the drivers in the roadside survey was approved by the Norwegian data inspectorate and the regional ethics committee. The study invitation and consent is described above.

## Results

### Participants

The case group was a sub-sample of a study including all patients admitted with injuries. Data were collected over a 12 month period from a large hospital emergency department (see Bogstrand et al., 2011 for further details). The case group consisted of 96 drivers admitted within six hours of injury (Table 2). The study of injured patients had no permission to collect data on non-participants. No information was therefore available on the distribution of drivers in the non- participant group. The refusal rate for the whole study was 7% and 24% were not invited to participate for other reasons described elsewhere [1]. The control group consisted of 5305 drivers, with a refusal rate of 6.2%. Six of the drivers who refused to participate were apprehended by the police before study inclusion because of positive alcohol breathalyzer test. There were no significant differences in gender distribution but there were more controls in the older (35–49 and ≥50 years) age groups and fewer in the youngest group (17–24). The difference in age distribution was statistically significant (p < 0.001). There were also statistically significant differences between cases and controls with respect to season and time and day (p <0.001) as shown in Table 2.

### Prevalence of psychoactive substances

Among all the injured drivers, 21.9% tested positive for one or more psychoactive substances. The substances most often found among injured drivers (case-group) were alcohol (11.5%), illicit stimulants such as amphetamines and cocaine (9.4%) and benzodiazepines (7.3%).

About three percent of the drivers in normal traffic tested positive for any substance. The most prevalent substances in moving traffic (control-group) was the hypnotic medicinal drug zopiclone (0.9%), the second was benzodiazepines (0.8%), and the third was cannabis (THC) (0.7%) (Table 1). Prevalence of psychoactive substances was higher in the case group than in the control group. Combinations of up to three or more individual medicinal or illicit drugs were present in both groups, as was alcohol, both alone and combined (Table 1). Combinations of drugs were more prevalent than single substance findings in the case group (11.5%) while single substance use of non-alcohol drugs was more prevalent in the control group (Table 1). Among the injured drivers, three of the four combinations with alcohol consisted of a stimulant drug (amphetamine, methamphetamine or cocaine) two of the cases was also combined with a hypnotic (zopiclone or flunitrazepam). Six of the seven cases who had combinations of non- alcohol drugs had combined benzodiazepines and stimulant drugs.

### Risk estimates

Crude and adjusted relative risk estimates (in terms of odds ratios) are presented in Table 3. The variables age, and time and day were differently distributed in cases and controls and they were also associated with the presence of alcohol alone or combined. Therefore, these variables were entered as co-variates in the multi-variate analyses of the association between presence of alcohol and injury risk. Age was the only co- variate associated with non-alcohol psychoactive substance use, and the only potential confounder entered in the multivariate analysis of non-alcohol psychoactive substance use and injury risk. Distribution of cases and controls differed significantly by time of accident or sampling time of control group. No significant association was found between the time variable and substance findings, the model was therefore not adjusted for time.

**Table 3:** Crude and adjusted odds ratio of accident risk (n = 5401)

***Alcohol***	***Crude OR (95% CI)***	***Adjusted OR (95% CI)***^***1***^
No alcohol (referent)		
Alcohol alone	29.0 (11.5- 73.0)**	36.1 (13.2- 98.6)**
Alcohol combined	124.4 (22.5- 688.5)**	231.9 (33.3- 1615.4)**
***Positive samples with no alcohol***	***Crude OR (95% CI)***	***Adjusted OR (95% CI)***^*2*^
No non-alcohol psychoactive substances (referent)		
One psychoactive substance	1.6 (0.5- 5.0)^(ns)^	1.4 (0.4- 4.4)^(ns)^
Two psychoactive substances	17.1 (5.6- 52.4)**	13.3 (4.2- 41.3)**
Three or more psychoactive substances	51.4 (11.3- 233.5)**	38.9 (8.2- 185.0)**

Chi-square test: ^(ns)^ = Not statistically significant, , ** = P < 0.001.

^1^Adjusted for age group and day and time.

^2^Adjusted for age group.

The analyses showed that alcohol was the only psychoactive substance that, when present alone, elevated the risk on non-fatal traffic injury statistically significantly (OR: 36.1, 95% CI: 13.2- 98.6, p < 0.001) (Table 3). The other two substances that were present alone among both cases and controls were THC and zopiclone. The prevalence of these substances alone was, however, very low in the case group, and no statistically significant association with risk of traffic injury was found. However, compared to drivers with no psychoactive substances present, the risk of non-fatal traffic injury was statistically significantly elevated among drivers with two non-alcohol psychoactive substances present (adjusted OR: 13.3, 95% CI: 4.2- 41.3, p < 0.001) (Table 3) and among those with three or more non-alcohol psychoactive substances present (adjusted OR: 38.9, 95% CI: 8.2- 185.0, p < 0.001) (Table 3). The highest OR for being injured in an accident was found for those who had both alcohol and one or more other psychoactive substances present (adjusted OR: 231.9, 95% CI: 33.3- 1615.4, p < 0.001) (Table 3).

## Discussion

This study revealed high risk estimates for alcohol and multi-drug use when comparing drivers admitted to the emergency department for injuries in car accidents with drivers in normal traffic from the hospital catchment area. The study found no statistically significant risk associated with the use of one non-alcohol psychoactive substance alone.

Other studies have also found significantly increased risk for accidents associated with the use of alcohol (see Taylor et al. for a review [27]). For instance, the Grand Rapids Study in 1964 found BAC levels above 0.04% to be associated with increased accident rates, and higher BAC levels gave higher accident rates [28]. Similar findings were published in another American study by Blomberg and colleagues in 2009 [29]. In the Netherlands, Movig and colleagues found that drivers who tested positive for alcohol (BAC above 0.8 g/l) had an increased likelihood of being injured in an accident; OR = 15.5 (95% CI:7.1- 33.9) [8] compared with 36.1 (95% CI:13.2- 98.6) in our study. The higher OR reflects a lower estimated prevalence of alcohol in the control group in our study. A previous study has shown that the prevalence of alcohol above the legal BAC limit in normal traffic is relatively low in Norway compared to other European countries [5], which is probably due to the restrictive BAC limits (0.2 g/kg) and related sanctions in Norway.

Single use of non-alcohol psychoactive substances was not statistically significantly associated with injury risk in our study, which is probably due to the low number of cases. With a larger case group, it might have been possible to identify psychoactive non- alcohol single substances associated with traffic accidents, as this was the most prevalent finding in the control group. In the present study, combinations of non- alcohol psychoactive drugs were more prevalent than single non-alcohol substances in the case group.

Prevalence of stimulant drugs was high in the case group. In other studies, prevalence of cocaine and amphetamines was low and no significant risk increase was found for single use of these substances [8,9]. Stimulant substances and amphetamines in particular are prevalent in Norwegian drivers apprehended by the police and the drugs are often found in combination with other psychoactive substances [30]. No stimulant substances was found alone in both the case and control group in this study so it was not possible to calculate an OR for the association between accident risk and single stimulant drugs.

The highest relative risk estimate in this study was for alcohol combined with one or more other psychoactive substances. Similar findings have been reported in earlier studies [7,8]. Reviewing the literature on the effect of cannabis compared with alcohol on driving Sewell and co-workers [31] concluded that combining alcohol with marijuana results in impairment even at doses which would be insignificant were they of either drug alone. Moreover, any combination of multiple psychoactive substances was associated with an increased risk of traffic injury. This was also reported by Gjerde and co-workers (2011).

### Strengths and limitations

A major strength of this study was the large control group. This enabled positive findings of different drugs and combinations even though the prevalence of psychoactive drug use among drivers in normal traffic was low. Another major strength was that analyses of the blood and oral fluid samples were performed by a laboratory accredited for analyzing psychoactive drugs in forensic toxicology since 1996. Equivalent cut-off thresholds for oral fluid and blood were used to compare the data, strengthening the risk estimates. Biological samples from cases and controls were analyzed for a wide spectrum of psychoactive substances, thereby allowing for a better assessment of combinations of psychoactive substances.

The number of cases was relatively low and consequently the test power for analyses of various specific substances and combinations of substances was low. Six drivers were apprehended by the police for drunk driving and refused to participate in the study; the OR for alcohol would have been somewhat lower if these drivers had been included in the analysis. On the other hand, blood samples were obtained up to six hours after the injury, which implies that some cases may be false negatives, in which case the relative risk estimate may be biased downwards.

The use of blood samples from controls would be better than oral fluid if the participation rate is high. However, we expected that using oral fluid with high participation rate was better than using blood with low participation rate. A limitation when using oral fluid was the uncertainty in determining cutoff concentrations in oral fluid that were equivalent to those in blood. In our study, we used those determined by the DRUID project, which so far are the best documented equivalent cutoffs. By using prevalence determinations with equivalent cutoff concentrations, the effect caused by inter-individual variations in oral fluid to blood ratios for drugs are reduced compared to using average OF/B ratios for estimations of drug concentrations in blood based on concentrations in oral fluid.

Driver culpability was not considered and risk estimates might have been higher for culpable drivers. There were some statistical differences in the distribution of age and time in the two groups but these differences were considered minor limitations. A major limitation was the unknown number of non-responders in the case group, and the relatively small case group. The authors have no data suggesting the numbers of non-respondents in the sub-sample of drivers or the direction of any possible bias.

### Implications

The high prevalence of alcohol in injured drivers and the significant increase in injury risk when driving under the influence of alcohol (whether alone or in combination with other substances) highlight the importance of curbing the extent of such driving. Various alcohol policy strategies are shown to be effective in reducing drunk driving and traffic crashes. These comprise not only low legal BAC limit and related sanctions [32,33] but also universal strategies as high taxes on alcoholic beverages [34] and high minimum legal age for drinking or purchase [35]. In Norway these effective strategies are employed to a larger extent than in most other OECD countries [36], and therefore other prevention strategies should also be considered. Multi-component programs with community mobilization [37] installation of ignition interlocks in drunk driving offenders’ vehicles [38] and mass media campaigns combined with high visibility enforcement [39] are strategies that are shown to be effective and therefore seem particularly relevant in this respect.

The latter type of strategy may also be relevant with respect to other substances than alcohol. An Australian study of ecstasy users found risk perception to be correlated with driving under the influence (DUI) of alcohol or other drugs [40]. The users who reported DUI did not perceive their driving to be impaired and believed that there was a low accident risk and a low risk of being apprehended by the police. Therefore, more information about the actual increase in injury risk with DUI and more visible enforcement may encourage individuals not to drive under the influence of psychoactive substances. Driving under the influence of alcohol or might be associated with alcohol dependence [41]. A review of studies on of interventions to reduce alcohol problems among ED- patients, found that interventions reduced alcohol related injuries [42]. Such interventions might be effective if they were offered to alcohol positive injured drivers.

The few single non-alcohol substances for which it was possible to calculate risk estimates, were not statistically significantly associated with accident risk, but combinations of two or more such substances were. Some of these substances are prescribed medicines and when prescribing more than one psychoactive medicinal drug the patient should be adequately informed about possible risks associated with multiple psychoactive substance use. Yet, it should also be noted that combined use of several psychoactive substances, even prescription drugs, may sometimes stem from illegal or informal sources.

Comparing oral fluid with blood samples gives more accurate estimates of present impairment compared to urine when using equivalent cut-off limits, and the use of oral fluid is, therefore, recommended in future studies. As combinations of different psychoactive substances were prevalent, one can not screen for just alcohol or just THC, for example, and calculate the risk for these substances. A full drug screen analysis is needed to ensure that a significant risk increase in one substance is not due to combinations with other substances.

### Conclusion

Prevalence of psychoactive substances was higher among injured drivers than drivers in normal moving traffic. Alcohol and stimulant drugs were particularly prevalent among drug positive injured drivers. The adjusted OR was high for alcohol combined with drugs; for alcohol alone, and for combinations of two or several non-alcohol substances. Various preventive measures should be considered to curb the prevalence of driving under the influence of psychoactive substances as these drivers constitute a significant risk for other road users as well as themselves.

## Competing interests

The authors declare that they have no competing interests.

## Authors’ contributions

STB contributed to the data collection of the case group, data analysis and drafted this manuscript. HG organized the data collection of the control group and analysis of biological samples. PTN organized the data collection of the case group and analysis of biological samples. All authors contributed to the design of the study and to the outline of the present paper, discussion of the results, the evaluation of the analytical results and data analysis, the writing of the manuscript. All authors approve the final version of the manuscript.

## Pre-publication history

The pre-publication history for this paper can be accessed here:

http://www.biomedcentral.com/1471-2458/12/734/prepub
